# Health risks of employees working in pesticide retail shops: An exploratory study

**DOI:** 10.4103/0019-5278.58914

**Published:** 2009-12

**Authors:** C. Kesavachandran, M. K. Pathak, M. Fareed, V. Bihari, N. Mathur, A. K. Srivastava

**Affiliations:** Epidemiology Division, Indian Institute of Toxicology Research, (Council of Scientific and Industrial Research), PB No. 80, Lucknow - 226 001, India

**Keywords:** Clinical studies, pesticides, shopkeepers

## Abstract

**Background:**

Shop keepers dealing with pesticides are exposed to multiple pesticides that include organophosphates, organochlorines, carbamates, pyrethroids. Hence an exploratory health study was conducted on shopkeepers selling pesticides in urban areas of Lucknow and Barabanki District, Uttar Pradesh, India.

**Materials and Methods:**

Detailed information regarding socio-economic status, family history, personal habits and work practices were recorded for 20 subjects and controls by the investigator on a pre-tested questionnaire. Clinical examination including neurological studies of the shopkeepers and control subjects was done.

**Results:**

The study revealed significant slowing of motor nerve conduction velocity and low peak expiratory flow rate among shopkeepers as compared to control subjects. Prevalence of significantly higher gastro-intestinal problems was also observed among exposed subjects. Neurological, ocular, cardiovascular and musculo-skeletal symptoms were also found to be higher among shopkeepers. This was not statistically significant. Significantly higher relative risk for sickness related to systems viz., cardio-vasular, genito-urinary, respiratory, nervous and dermal was observed among exposed subjects compared to controls.

**Conclusions:**

These findings provide a prima facie evidence of clinical manifestations because of multiple exposures to pesticides and poor safety culture at work place.

## INTRODUCTION

Pesticide retail shops are localized in the urban areas and cater to the growing demands of urban population for house hold products used in lawn and gardens. These include insect sprays and baits, disinfectants, rat poison. Pesticides are also used to control various pests in the areas of public health, livestock and agriculture. Registered pesticides of different varieties are also available to tackle the problem of pests and vectors. Pesticides are usually sold to consumers by private retailers and/or wholesalers in India. In some cases they are also distributed by the Ministry of Agriculture. India is the largest manufacturer of basic pesticides in Asia and ranks 12^th^ globally. Insecticides account for 75% of India's total pesticide consumption, followed by fungicides (at 12%) and herbicides (at 10%).[1]

The reports addressing the health concerns of pesticide exposure are confined to either incidental/accidental[2–4] or occupational poisoning.[5–11] Workers in the pesticide manufacturing industry, pesticide shop keepers and users of pesticide for agricultural and other public health activities are all exposed to pesticides. Pesticide retailers are an important link in pesticide distribution chain in India. Implementation of mandatory use of Personal Protective Equipment (PPE) among workers in India is very slack. Further, because of huge population, this group constitutes a sizable number of subjects in India. There is no literature with respect to health problems among shop keepers occupationally exposed to pesticides in India and other developing countries. The distribution of pesticides for agricultural purposes is mainly through retail shops and hence such shopkeepers are exposed to a mixture of pesticides in India and many other developing countries. The highlights of the present study can be utilised to decide upon the following:

1. Need to protect such occupations,
2. Under take detailed studies for eliciting the specific issues,
3. Finally formulating and implementing regulatory guidelines in pesticide retail marketing.

The present study is an attempt to explore the health problems of multiple pesticide exposures among pesticide shopkeepers in urban areas.

## MATERIALS AND METHODS

### Study design and study subjects

A cross sectional study design was employed in the study. Twenty shops located in urban areas of Barabanki and Lucknow district were randomly selected so that a proportional representative sample of pesticide shops of two districts is covered in the study. List of retail pesticide outlets in the district were taken and random number Tables used for selection. All the 32 male (no female were employed) subjects employed in retail shops were included as exposed group. The study was conducted during March to June 2008.Twelve of the exposed subjects did not give consent for the study and only 20 could be studied in the exposed group. Eighteen subjects of similar socioeconomic status who did not handle pesticides were studied as control group. The control group comprised of employees working mainly in administrative offices in the same area.

None of the study subjects was suffering from any chronic disease like tuberculosis, diabetes, thyroid malfunction or malignancy during clinical examination. This was necessary to rule out the confounding influence of diabetes especially in nerve conduction studies.[3,7] No study subject suffered from a major head injury which required hospitalization of more than a week.

### Collection of information

Detailed information regarding socioeconomic status, family history, type of physical activity, personal habits were recorded for each subject on a pre-tested questionnaire. The questionnaire was in Hindi (local language) and entered the information in questionnaire by the investigators.

Work practices of retailers like use of protective devices, bathing practices after closing the shop, awareness of pesticide toxicity, exhaust fan facility at pesticide storage place, cleaning practices in case of spillage during loading and unloading process were recorded using questionnaire.

### Ethical issues

The purpose of the study was explained to all the participants and their consent was obtained. The requisite clearance of institutional human ethics committee was obtained from Indian Institute of Toxicology Research, Lucknow, India for the study.

#### Medical examination

Clinical examination of all the 20 exposed subjects selling pesticides and 18 controls was done. The clinical examination comprised medical history, general physical examination and detailed examination of the central nervous (CNS), respiratory (RS), cardiovascular (CVS), gastro-intestinal (GIS), ocular, dermal and musculoskeletal (MSD), reproductive (RPS) and genito-urinary systems (GUS). Signs and symptoms observed in each subject were recorded in the questionnaire.

#### Lung function studies

Lung function test were performed using portable Spirometer (Sibelmed, Italy). Peak Expiratory Flow Rate (PEFR), Forced Expiratory Volume in 1 sec (FEV_1_) and Forced Vital Capacity (FVC) were measured. Spirometric data were transformed into percentages based on reference values,[12] calculated in relation to sex, age, weight and height. A minimum of three measurements were conducted for each test per subject and the highest value was selected for the analysis.

#### Nerve conduction velocity studies

Nerve conduction studies were performed on EMG-EP electromyography (RMS, India).Subjects were allowed to acclimatize in air conditioned room (25°C) for 15 minutes before the procedure. Recordings were obtained at following instrument settings: For motor studies: Sensitivity: 2-5 mv/ mm, low frequency filter: 2-5 Hz, high frequency filters: 10 KHz, sweep speed; 1-2 ms/mm. For sensory studies: Sensitivity: 10-20 μv/mm, low frequency filter: 2-3 KHz, sweep speed: 1-2 ms/mm. Stimulation was done using standard supra maximal technique using a square wave of 0.1 ms duration. Distance was measured using a metal tape. Motor (MNCV) as well as sensory nerve conduction velocity (SNCV) of median nerve of left forearm was measured. Latency was measured as time interval between stimulus artefact and onset of electrical response. Nerve conduction velocity was calculated by dividing the latent period by nerve length. Motor nerve conduction velocity (MNCV) and sensory nerve conduction velocity (SNCV) were measured using the protocol proposed by Kimura.[13] Electro diagnostic reference value suggested by Kimura *et al*.,[13] were considered for the normal values for the MNCV and SNCV and lower values from the normal value considered as low MNCV and SNCV. Subjects with clinically suspected peripheral neurological abnormalities were tested for nerve conduction velocity.

### Statistical analysis

The statistical significance of mean values of different parameters (age, height, weight, FVC, FEV_1_, PEFR, MNCV, SNCV) in exposed and control were tested using student's ‘t’ test after ascertaining the homogeneity of variance between the two groups using variance ratio test. Significance of prevalence of various disorders in exposed, as compared to control subjects, was tested using Chi square test where expected cell frequencies were more than 5. Fisher exact test was applied in situations where expected cell frequencies were less than 5. MS Excel was used for statistical analysis with the help of a statistician (NM).

## RESULTS

**Figure 1:**
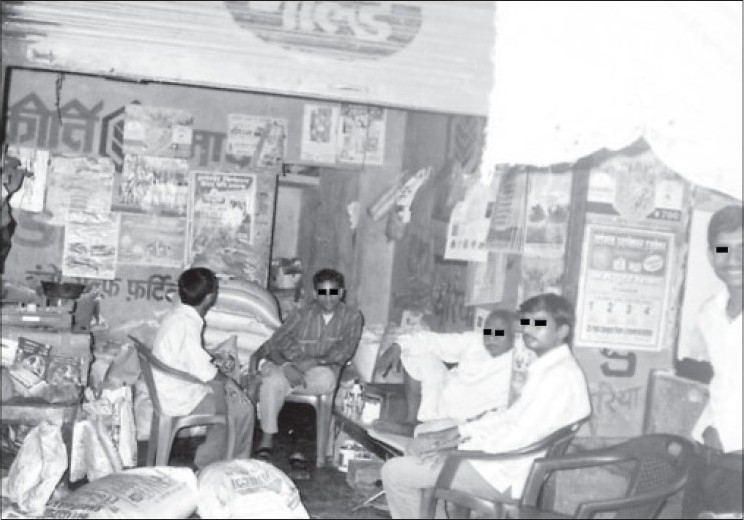
Show rooms with ware house in a shop

**Figure 2:**
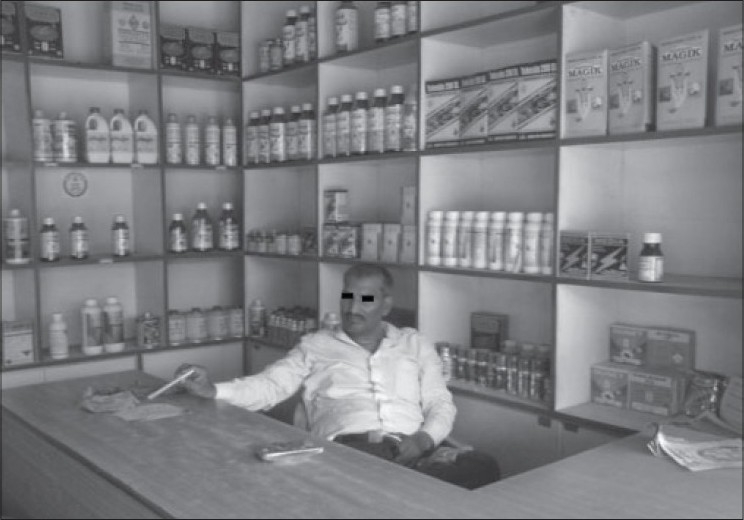
Show rooms of pesticide shops

**Figure 3-4:**
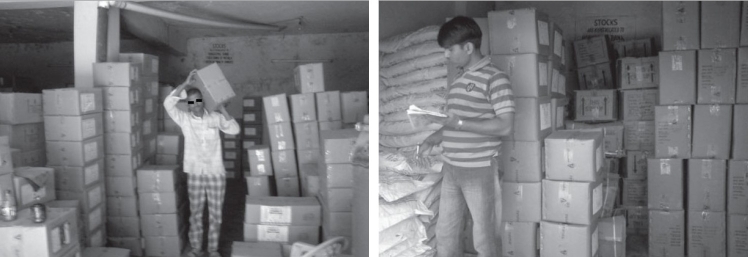
Ware house of pesticide shops

Pesticides of different brands comprising organophosphates, organochlorines, carbamates, pyrethroids, fumigants were stocked in the shops. Pesticide shops generally have a show room and a ware house. Limited quantities of pesticides are displayed in showroom. Packaged products available in plastic packets, bottles, tin etc are kept in different racks [Figures 1–2]. In a ware house, large quantities of pesticides are just dumped on the floor [Figures 3–4]. Ware houses have poor ventilation with just one door opening towards the show room. Some units have both showroom and ware house in a single shop [Figures 3–4]. Pesticide exposure is higher in warehouses compared to showrooms since warehouses have no exhaust facility. Ten per cent of study subjects were owners of the shop and sit in the showrooms while rest of the subjects move between ware house and showrooms in course of their work.

Physical characteristics, personal habits, occupational details etc of exposed and study subjects are shown in Table 1. Age, height and weight of retailers and controls were similar (> 0.05). Personal habits like alcohol consumption, cigarette smoking, tobacco chewing, pan chewing were observed among retail shop keepers and its percentage distribution was statistically similar with that of controls.

**Table 1:** Physical characteristics and other details of pesticides retailers

Parameters	Control (n = 18) Mean ± SD	Retailers (n = 20) Mean ± SD	*P* value
Age (yr)	32 ± 7.0	34.21 ± 9.8	> 0.05
Height (cm)	169.14 ± 6.7	170.85 ± 5.0	> 0.05
Weight (Kg)	62.7 ± 8.3	61.77 ± 8.5	> 0.05
Yrs of exposure	Nil	15 ± 12.8	
Type			
Alcoholic consumption n(%)	2 (11.1)	3 (15)	>1.00
Cigarette smokers n (%)	3 (16.7)	6 (30)	>0.45
Tobacco chewers n(%)	2 (11.1)	3 (15)	>1.00
Pan chewers n(%)	1 (5.5)	5 (25)	>0.18
Education			
Illiterate n (%)	1 (5.5)		
Primary n (%)	2 (11.1)		
Middle n (%)	6 (33.3)	3 (15)	
High school n (%)	4 (22.2)	2 (10)	
Intermediate n (%)	1 (5.5)	4 (20)	
Graduate n (%)	3 (16.7)	10 (50)	
Post graduation n (%)	1 (5.5)	1(5)	

Protective devices were not used by any of the exposed subject. However, all shopkeepers are aware of pesticide toxicity. The assessment of awareness was made by asking relevant questions related to pesticide toxicity signs and labels, cautions mentioned in each pesticide. Shopkeepers spend at least four hours/day in closed environments. The workers eat, drink and smoke in the workplace. They take bath in the evening after the business hours of pesticide sales and do not wash hands after each sale of pesticide to customer. Loading and unloading of packets from the cartons and its transport from ware house and showroom is done by bare hands and if load is heavy, they carry them on shoulders or the head. Safety practices for clean up and protection were recorded using questionnaire and none of the shopkeepers followed any clean up, protection practices. None of the shops studied had exhaust fan facility in ware houses. All the shopkeepers admit that spilling of pesticides on the floor occurs during loading and unloading process.

**Figure 5:**
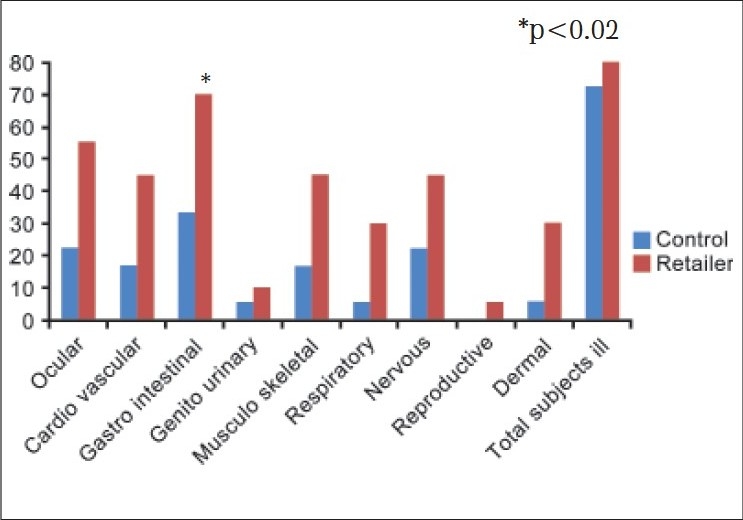
Morbidity profile of retail pesticide shop keepers

Pesticide retail shopkeepers show higher morbidity than controls [Figure 5]. Seventy per cent shopkeepers show significantly higher prevalence of gastro-intestinal problems. Subjects with symptoms were also higher among shopkeepers as compared to controls. Ocular problems were higher (55%) among shop keepers as compared to control. Cardiovascular abnormalities were observed in forty five per cent shop keepers. Forty five per cent shop keepers suffer from musculo-skeletal symptoms. Nervous system abnormalities were observed among 45% shopkeepers. Gastro-intestinal symptoms observed were hyperacidity, burning sensation in abdomen, pain in abdomen, vomiting, constipation and jaundice. Gastro intestinal problems were significantly higher among retail shop keepers compared to control. Ocular problems include watering of eyes, pain in eye, irritation of eyes and red swollen eye. Nervous system abnormalities observed were headache, tingling/numbness, changes in smell/taste, fatigue, forgetfulness, tremors, frequent fainting and shooting pain in limbs. Musculo-skeletal disorders like swollen and painful joints, pain in body, muscle twitching, weakness in arms and legs were also observed among exposed subjects. Dermal problems observed were warm and burning sensation of skin, itching.

Cardio-vascular problems include cases of hypertension. Morbidity profile shown in Figure 5 is without controlling any specific confounders, due to small sample size. An attempt was made by ruling out the chronic illness among subjects. We observed that the pesticide shopkeepers were at a higher risk for sickness including ocular problems (RR-2.16; 95% CL:0.83-5.62; *P* < 0.15), cardiovascular problems (RR-3.36; 95% CL:1.13-9.99; *P* < 0.02), gastro-intestinal problems (RR-1.92; 95% CL:0.94-3.93; *P* < 0.09), genito-urinary problems (RR-8.64; 95% CL:1.23-60.61; *P* < 0.007), musculo-skeletal problems (RR-2.88; 95% CL:0.95-8.74; *P* < 0.07), respiratory problems (RR-8.64; 95% CL:1.23-60.61; *P* < 0.007), nervous system related problems (RR-2.88; 95% CL:1.16-7.18; *P* < 0.016), problems related to reproductive system (RR-4.32; 95% CL:0.57-32.84; *P* < 0.21), skin (RR-4.32; 95% CL:1.34-15.24; *P* < 0.004).

Low FVC was observed in five shopkeepers compared to three controls. Low FEV_1_ was observed in 10 exposed subjects compared to six controls. Low PEFR was observed in three exposed subjects compared to two controls subjects. Significantly low mean value of PEFR (L/min) was observed in exposed (330 ± 183.08) as compared to control (457.14 ± 100.91) [Table 2]. Mean values of FVC (L) and FEV_1_ (L) were 2.53 ± 0.32 and 2.57 ± 0.71 respectively compared to controls (FVC: 2.84 ± 0.81 and FEV1: 2.04 ± 0.58). Mean FEV_1_ was significantly low in controls compared to exposed subjects.

**Table 2:** Lung function status of pesticide retail shopkeepers

Parameters	Control (n = 18)	Retailers (n = 14)	*P* value
FVC (L)			
Mean ± SD	2.84 ± 0.81	2.53 ± 0.32	0.20 < *P* < 0.10
Range	1.34-4.33	2.07-3.04	
Low: N (%)	3 (16.66)	5 (35.71)	0.2516 NS
FEV_1_ (L/S)			
Mean ± SD	2.04 ± 0.58	2.57 ± 0.71	0.02 < *P* < 0.05
Range	0.97-3.12	0.82-3.49	
Low: N (%)	6 (33.33)	10 (71.42)	0.0748 NS
PEFR (L/min)			
Mean ± SD	457.14 ± 100.91	330 ± 183.08	0.01 < *P* < 0.02
Range	206-548	356-480	
Low: N (%)	2 (16.6)	3 (21.42)	0.6312 NS

Table 3 shows observation of nerve conduction velocity of median nerve in shop keepers. NCV was conducted on nine shop keepers and four control subjects. These Subjects were clinically suspected to be suffering from peripheral neurological abnormalities. The subjects were found to have one of the following sign/symptom like numbness and tingling of arm, muscle weakness, paresthesia and pain in upper limbs. Low MNCV of median nerve as per electro-diagnostic reference value was observed in six out of nine shop keepers. Low SNCV as per electro-diagnostic reference value of median nerve was found in one out of nine shopkeepers.

**Table 3:** Nerve conduction studies of pesticide retail shopkeepers

MNCV (m/sec)	Control (n = 4)	Retailers (n = 9)	*P* value
Mean ± SD	50.24 ± 10.74	32.88 ± 15.72	*P* < 0.05
Range	28.79-54.79	19.23-57.69	
Low: N*	2	6	1.00 NS
SNCV (m/sec)			
Mean ± SD	52.56 ± 8.36	52.56 ± 8.98	1.00 NS
Range	38.46-65.66	34.65-63.73	
Low: N*	1	1	1.00 NS

* Out of nine retailers and four controls medically diagnosed for peripheral neurological abnormalities

Lower mean value of MNCV (32.88 ± 15.72 m/s) were observed in exposed as compared to control (50.24 ± 10.74 m/s) [Table 3]. However, statistically significant difference in mean values of SNCV among exposed (52.56 ± 8.98 m/s) and control (52.56 ± 8.36 m/s) subjects were not observed in the present study.

## DISCUSSION

The present study shows that retail shop keepers are exposed to various pesticides comprising organophosphates, organochlorines, carbamates, pyrethroids, fumigants in the course of the work. The exposure occurs through ingestion, inhalation and dermal contact. These persons do not use PPE. Non-use of PPE is attributed to poor enforcement, legislative requirements with respect to safe and proper handling. The exposure occurs during spillage, loading, unloading, keeping pesticides in rack etc. Factors like non-use of personal protective equipment, work practices related to hygiene, spills, and attitudes toward risk may all influence the degree of pesticide exposure and can be incorporated into exposure estimates but the relationship of these factors to exposure is complex.[14]

High prevalence of symptoms related to nervous system viz., headache, changes in smell/taste, fatigue, forgetfulness, tremors, frequent fainting, pain in limbs were observed among shop keepers. Symptom prevalence like headache, dizziness, fatigue, insomnia, nausea, chest tightness, and difficulty in breathing as well as symptoms suggesting cognitive (confusion, difficulty concentrating), motor (weakness, tremor), and sensory (numbness, tingling, visual disturbance) dysfunction have been reported in pesticide exposed subjects in earlier report.[14] One study suggested that the sense of smell was not affected by OP;[15] another study suggested a relationship with fumigants.[16] Tremor was related to exposure to multiple pesticides in an earlier report;[14] tremor was not observed due to OP exposure in another report.[15]

Nerve conduction studies show significant decline in MNCV among those studied. Nerve conduction velocity is a sensitive indicator of the effect of pesticides on peripheral nervous system.[13] Reduced nerve conduction velocity was also observed in an earlier study in organo phosphate exposed workers[17] and fumigators.[16] Contrary to this OP exposure showed little evidence of impaired nerve conduction among exposed subjects.[14,15] Organophosphorus compounds readily cross the blood brain barrier which results in an excess of available acetylcholine at particular central nervous system receptors and at neuro-muscular junctions by inhibiting cholinesterase. The neurotoxic effects of exposure to organophosphorus compounds occur in three successive clinical stages viz., acute cholinergic crisis, intermediate syndrome and organophosphate induced delayed peripheral neuropathy.[18] The chemicals involved in these distinctive intoxications include fenthion, dimethoate, monocrotophos and methamidophos.[13]

Gastro-intestinal system viz., hyperacidity, burning sensation in abdomen, pain in abdomen, vomiting, constipation, jaundice were significantly higher among exposed shop keepers as compared to control. Gastro intestinal problems among pesticide sprayers have also been reported by other investigators.[9,19] Pesticides can enter the body through the mouth (also called ingestion). This can occur when hands are not properly washed before eating food. Ingested materials can be absorbed anywhere along the gastrointestinal tract; the major absorption site is the small intestine. Once absorbed, they eventually enter the blood stream by one of several means, and circulate throughout the body. Musculo-skeletal disorders like joints painfully swollen, pain in body, muscle twitching, weakness in arms and legs were also observed in exposed shop keepers. An earlier study also found musculo-skeletal disorders to the tune of 15% among pesticide sprayers.[20] Musculo-skeletal problems observed among subjects may be due to wrong ergonomic practices in course of loading and unloading of pesticide containers etc.

Dermal problems like warm and burning sensation of skin, itching were observed in arms of retailer shop keepers. Ocular problems among pesticide sprayers reported in earlier reports.[9,20] include watering of eye, pain in eye, red swollen eye, irritation of eye etc. Exposure of unprotected eyes to pesticides results in the absorption in ocular tissue and potential ocular toxicity.[21] Ocular pesticide exposure involves the direct entry of these chemicals into the eye tissue. These exposures could also occur from accidental splashes of these chemicals that enter the eye. As a result, these chemicals are absorbed through the eye tissue and enter the circulation.[21]

Mean values of lung functions, FVC and PEFR showed significant reduction in exposed subjects compared to control. Lung function abnormalities viz., low FVC, FEV_1_, PEFR observed among pesticide retailers may be due to work related exposure to pesticides. Fumigants, primarily methyl bromide and sulfur dioxide, can cause a severe acute pulmonary response in occupationally exposed agrarian subjects.[22] Paraquat, a herbicide, is the only individual pesticide that has been studied with respect to respiratory disease in population based settings. Paraquat was associated with increased wheeze among Nicaraguan banana workers[23] and decreased lung function among South African farm workers.[24] Earlier studies of our group also reported lung function decline in pesticide sprayers.[8,25] A significantly low mean FEV_1_ was observed in control group compared to exposed group and reasons for the same is unknown.

Small sample size and lack of objective data with respect to blood levels of pesticides makes it imperative that a more detailed epidemiological study be undertaken to elucidate the effects of pesticide on the health of subjects exposed during retail and whole sale marketing of pesticides.

## CONCLUSION

To the best of our knowledge, this is the first report of ill effects by multiple pesticide exposure among subjects employed in retail pesticide outlets. Significant relative risk for sickness related to systems viz., cardio-vasular, genito-urinary, respiratory, nervous and dermal was observed among exposed subjects. The study also revealed a compromised lung function status evidenced by low PEFR. The nerve conduction among the exposed subjects was also found to be impaired. This was reflected in low values of MNCV among shopkeepers as compared to control subjects. These findings are in consonance with earlier reports on pesticide toxicity.

Sound national policies are needed to effectively articulate appropriate guidelines for reducing pesticide exposure at work place (shops). It is recommended that detailed studies to formulate an action plan for promoting the health of this group should be urgently undertaken.
